# When HIPAA hurts: legal barriers to texting may reinforce healthcare disparities and disenfranchise vulnerable patients

**DOI:** 10.1038/s41372-024-02080-5

**Published:** 2024-08-15

**Authors:** Denita Lindsey, Rachel Sinkey, Colm Travers, Henna Budhwani, Molly Richardson, Rosylen Quinney, Janet M. Turan, Eric Wallace, Martha S. Wingate, Alan Tita, Waldemar A. Carlo, Vivek V. Shukla

**Affiliations:** 1 University of Alabama at Birmingham, Birmingham, AL, USA; 2 Florida State University, Tallahassee, FL, USA

## Abstract

Effective health communication between healthcare providers and patients is a cornerstone of quality healthcare. It underpins trust, comprehension, and patient-informed care. Robust research shows that effective communication, including the use of text messaging for communication can improve maternal/fetal and neonatal outcomes and patient satisfaction, particularly among vulnerable patients. Health information privacy laws that do not evolve with technological advances can inadvertently create barriers to effective health communication, reinforcing perinatal disparities. This is particularly true regarding maternal and child health, where the use of text messaging for patient communication has the potential to make a substantial impact on health disparities. This article explores the complex interplay between health information privacy laws and text messaging, highlighting challenges and examining potential solutions. It stresses the need for consistent health information privacy laws that protect the privacy security of health information for pregnant patients and new mothers, while also aligning with evolving communication technologies.

The World Health Organization recognizes and supports digital communication, including text messages, as an intervention to reduce inequities and improve maternal, newborn, and child health behaviors [1]. The value of text messaging communication in healthcare is substantial. Recent studies indicate that text messaging is one of the most common forms of global communication, with widespread accessibility due to the omnipresence of text-enabled cell phones in high-income as well as low- and middle-income countries. Text messaging is cost-effective and provides a convenient and less intimidating platform for patients and providers to engage in real-time interactions, eliminating the need to step away from daily responsibilities and not requiring access to the internet or a smartphone.

Effective health communication is a conduit to build trust, ensure comprehension, and solicit patients’ perspectives and concerns, enabling patient-informed healthcare [2]. A robust body of research underscores the positive impact of effective health communication on patient outcomes, adherence to treatment plans, patient satisfaction, and reduction of disparities [3–5]. Additionally, ensuring health information confidentiality is needed to build trust and promote open provider-patient communication to improve healthcare and health outcomes. Health information privacy laws serve as essential standards to ensure the security of patient health data. These health information privacy laws, including the Health Insurance Portability and Accountability Act (HIPAA) and other federal and state laws, have been established to safeguard the confidentiality and security of patient privacy in the United States (US). Nevertheless, the interaction between these laws and the dynamics of health communication is intricate and can sometimes unintentionally introduce barriers to healthcare. In this article, we explore the complex relationship between health information privacy laws and health communication. We highlight the issues and examine potential solutions to mitigate these barriers, specifically in reference to text messaging as a form of health communication, in an effort to help improve healthcare outcomes, patient experiences, and reduce perinatal health disparities.

Recent technological advances enabled a profound shift in communication modalities and patterns, especially among younger generations who have grown up using digital technology. The ubiquity of smartphones, social media platforms, and instant messaging has revolutionized how people connect, share information, and collaborate. Communication has become faster and more convenient, bridging geographical distances and fostering seamless electronic interactions. This transformation extends to healthcare, where patients increasingly expect the same level of convenience and connectivity with their healthcare providers [6]. The landscape of healthcare communication is transforming with newer technologies like electronic health records, telemedicine, and mobile health apps that facilitate real-time information sharing, remote consultations, and seamless access to medical records [7]. These newer technologies are relevant to Healthy People 2030 objective HC/HIT-07 to increase the proportion of adults who use information technology to track health care data or communicate with providers [8].

Recent data suggest that women who are currently of childbearing age prefer text-based healthcare communications [9]. Many healthcare organizations have integrated texting to enhance provider-patient communication, particularly in low- and middle-income countries, where digital health interventions have shown compelling benefits for pregnancy, postpartum, and postnatal care [10]. Emerging evidence from US studies suggests that text-based interventions, such as appointment reminders and health literacy information, for pregnant people with social risk factors can improve health behavior and potentially help address health disparities [11–13].

Increasing numbers of hospitals and hospital systems are using internet-based communication and health care tools. However, there are pronounced disparities in home broadband service access by race/ethnicity, age, geographic location, education, and income [14]. There have been strategies advocated to increase broadband internet access to improve health, yet progress on this objective is declining [8]. Text messaging, on the other hand, is almost universally accessible, affordable alternative for health communication that does not require internet access. This makes text messaging particularly valuable for socially disadvantaged groups. The ease of use and low cost associated with text messaging position it as a promising approach for improving access to care, especially in marginalized communities lacking internet access or smartphones. Frequent interactions through text messaging may enhance motivation, education, and behavior change opportunities. It can potentially be employed to send appointment reminders, stress the significance of follow-ups, and encourage wellness check-ins, potentially encouraging patients to seek necessary medical care more promptly. Additionally, personalized text messaging interactions can offer timely, practical suggestions and easily accessible resources, thus empowering patients to take a more active role in managing their health effectively [15, 16].

However, text messaging is not considered a secure form of communication, given it is not secure at rest, meaning, some mobile devices used to access text messages may not have access controls in place, such as strong passwords or facial recognition, making it easier for a person other than the intended recipient to intercept the transmitted information. Thus, despite the emerging evidence with potential positive outcomes regarding text-based interventions, text messaging’s compliance with HIPPA’s security rule remains contingent on institutional interpretation. We recently encountered challenges arising from US privacy laws, with using text-based digital health interventions while developing a randomized clinical trial to improve maternal and neonatal outcomes in marginalized communities (P3 Providing an Optimized and emPowered Pregnancy for You, POPPY project, American Heart Association, 22HERNPMI985239;clinicaltrials.gov identifier NCT05940688). The Security Rule mandates that healthcare organizations/providers ensure the confidentiality and integrity of electronic personal health information while protecting against anticipated threats to security and prohibited uses. Compliance with the Security Rule becomes the institution’s responsibility, demanding that institutions assess their specific needs and develop strategies that meet their objectives without exposing them to legal liabilities, such as those that could potentially be encountered by sending insecure text messages, including health-related information. To reduce liabilities and comply with privacy law requirements, and the institutional policy we were required to change our approach from conventional text message communication to using a secure web link for message transmission. Although this approach ensured higher data security and privacy law compliance, it created a new challenge; it limited accessibility for patients from marginalized communities who lacked resources for smart phones and reliable internet access. Further, the additional “clicks” required to open the secure browser appeared to reduce engagement with the intervention. The secure web link for the message transmission system made the overall intervention less accessible and less inclusive, potentially excluding the most vulnerable patients and risking healthcare disparities. These conflicting issues lead to a paradox where the existing laws, rather than enabling health communication, may in fact, hinder it. This issue likely affects other institutions, providers, and researchers in the US [9].

Initially enacted in 1996, HIPAA was established to protect the privacy and security of patients’ personal health information in the US. The Privacy Act of 1974 regulated personal information held by federal agencies, laying the foundation for the privacy components of HIPAA. Similarly, the Computer Security Act of 1987 introduced security standards for federal computer systems, which formed the basis for HIPAA’s later emphasis on safeguarding electronic health records. HIPAA has undergone substantial amendments over the years. The Privacy Rule, enacted in 2000, established standards for protecting individuals’ medical records and personal health information [17]. The Security Rule, introduced in 2003, delved into the intricacies of electronic health information security, recognizing the growing role of technology in healthcare [18]. The Health Information Technology for Economic and Clinical Health (HITECH) Act of 2009 was pivotal in expanding HIPAA’s scope by intensifying penalties for noncompliance and encouraging the widespread adoption of electronic health records [19, 20]. HIPAA’s ongoing evolution reflects its adaptability to a changing healthcare landscape, ensuring its continued relevance and effectiveness in safeguarding patient privacy. HIPAA’s significance extends beyond protecting patient privacy. Due to its rigorous enforcement and the associated penalties for non-compliance, HIPAA profoundly influences virtually every facet of healthcare delivery and, consequently, health communication [21, 22]. In addition to HIPAA, other health information privacy laws have been established at state and federal levels. State and federal health information privacy laws are often complementary but can also have inconsistencies, leading to variable interpretations among institutions and agencies.

Striking the right balance between patient privacy and effective communication is a formidable challenge, and diverse arguments emerge from both sides [23]. Patient privacy is sacrosanct, and health information privacy laws endeavor to protect it; on the other hand, effective health communication and information exchange is indispensable for optimal healthcare. The need to comply with health privacy laws can create communication barriers within the healthcare system, hindering provider-patient dialogue instead of facilitating it [24, 25]. The fragmented nature of health information privacy regulations interpretation and regulatory complexity in the US impacts patient care and delays treatment and therapy development, affecting care quality and patient outcomes while hindering researchers from exploring innovative interventions and fostering a risk-averse culture that may stall medical progress.

We reason that establishing unambiguous health information privacy laws devoid of interpretation discrepancies is imperative, ensuring generalizability, replicability, and consistency in application across the states and institutions within the US. Fostering an effective and equitable healthcare environment requires thoughtfully addressing the health information privacy laws–health communication and information exchange paradox to fully leverage the potential of text messaging communication and other digital health interventions [15, 16]. The solution likely lies in streamlined and patient-preferred adaptable privacy laws that protect patient privacy while allowing text message communication in the evolving digital healthcare landscape [26].

Furthermore, healthcare professionals or administrators should receive training on privacy laws, data ethics, and health communication and information exchange documentation. Sharing success stories and best practices from healthcare organizations that have successfully balanced health information privacy laws and effective communication can serve as valuable templates for other institutes and researchers navigating similar challenges. Promoting patient education about their rights under health information privacy laws is also crucial. Patients should understand the purpose of these regulations and how they can access, control, and share their healthcare information. Actively involving patients in decision-making processes regarding sharing their healthcare data is paramount. Empowered patients can provide informed consent and express preferences, allowing them to select a preferred form of communication on a somewhat less secure platform. Equipping patients with resources and strategies to address health information privacy laws-related concerns, such as accessing their medical records, filing complaints, and seeking clarity on their privacy options, empowers them to actively manage their health data.

Looking ahead, several areas merit further research and policy development. There is a need to explore advancements and research in cost-effective and accessible health communication technologies for patients from marginalized communities, ideally with seamless integration into medical records to facilitate tracking and audit. Moreover, it is essential to advocate for ongoing dialogue among key stakeholders, including patients, healthcare providers, and policymakers, implementing regular policy updates to ensure that health information privacy laws remain aligned with the evolving communication and healthcare landscape. Such a process will ensure the relevance of these laws to protect patient privacy, empower patients in managing their health data, and facilitate effective communication, particularly for patients at risk for adverse health outcomes and disparities. By implementing technological solutions, amending standard policies, and adhering to best practices, the healthcare community can navigate the complex landscape of health information privacy laws while prioritizing both patient privacy and effective communication.

In conclusion, this article highlights the intricate relationship between health information privacy laws and health communication, and underscores how well-intentioned regulations can sometimes impede effective communication. It emphasizes the need for unambiguous health information privacy regulation that eliminates interpretation discrepancies, allowing a more balanced approach that prioritizes patient care as well as patient autonomy. This approach may enable more effective healthcare communication, implementation of, and access to evidence-based interventions that translate into improved maternal and child health outcomes.
